# Hermaphroditism in *Cannabis sativa* L.: Impacts, Inducers, and Industry Implications

**DOI:** 10.3390/plants15111643

**Published:** 2026-05-27

**Authors:** Chaylen Douglas Richards, Byeong-Ryeol Ryu, Gyeong-Ju Gim, Sang-Hyuck Park

**Affiliations:** Institute of Cannabis Research, Colorado State University Pueblo, Pueblo, CO 81001, USA; chaylen.richards@csupueblo.edu (C.D.R.); byeongryeol.ryu@csupueblo.edu (B.-R.R.); gyeongju.gim@csupueblo.edu (G.-J.G.)

**Keywords:** *Cannabis sativa* L., hemp, hermaphroditism, sexual plasticity, monoecious, dioecious

## Abstract

*Cannabis sativa* L. is a predominantly dioecious species, but sex expression is highly plastic and can be modified by genetic, hormonal, developmental, and environmental factors. This plasticity has major implications for commercial production because hermaphroditic expression in female plants can cause unintended pollination, seed formation, reduced floral quality, and losses in cannabinoid yield. This review summarizes current understanding of sex determination and sex-expression instability in *C. sativa*, with emphasis on hermaphroditism and its agronomic significance. We examine the genomic architecture of sex determination, the roles of ethylene and gibberellin signaling, and the effects of exogenous chemical treatments used to alter sexual phenotype. Particular attention is given to silver-based ethylene inhibitors, especially silver thiosulfate, which remain the most effective tools for induced masculinization and feminized seed production. We also assess the role of environmental stressors in sex instability and review current approaches for early detection, including visual inspection, Raman spectroscopy, and sex-linked molecular markers. Overall, the available evidence supports a multilayered and context-dependent model in which genotype, treatment regime, developmental stage, and environmental conditions interact to shape sexual phenotype. Improved understanding of these processes will be essential for reducing hermaphroditic risk, improving breeding strategies, and supporting stable, high-value cannabis production.

## 1. Introduction

*Cannabis sativa* L. has reemerged as a high-value agricultural and medicinal crop due to increasing interest in its diverse phytocannabinoid profile, including delta-9 tetrahydrocannabinol (Δ^9^-THC) and cannabidiol (CBD), among more than 150 identified cannabinoids [1]. Glandular trichomes, the primary sites of cannabinoid biosynthesis and accumulation, are especially abundant on unpollinated female inflorescences (commonly referred to as sinsemilla in industry) [2]. Because these compounds are concentrated in glandular trichomes associated with unfertilized female flowers, reproductive stability directly affects the quality and value of cannabinoid-focused crops [3]. With increasing legal acceptance of recreational, medical, and industrial cannabis across multiple jurisdictions, the U.S. cannabis market has been projected to reach $50.7 billion in sales by 2028 [4]. Consequently, traits influencing floral development, cannabinoid production, and reproductive stability have become central targets in cannabis research and breeding.

**Figure 1:**
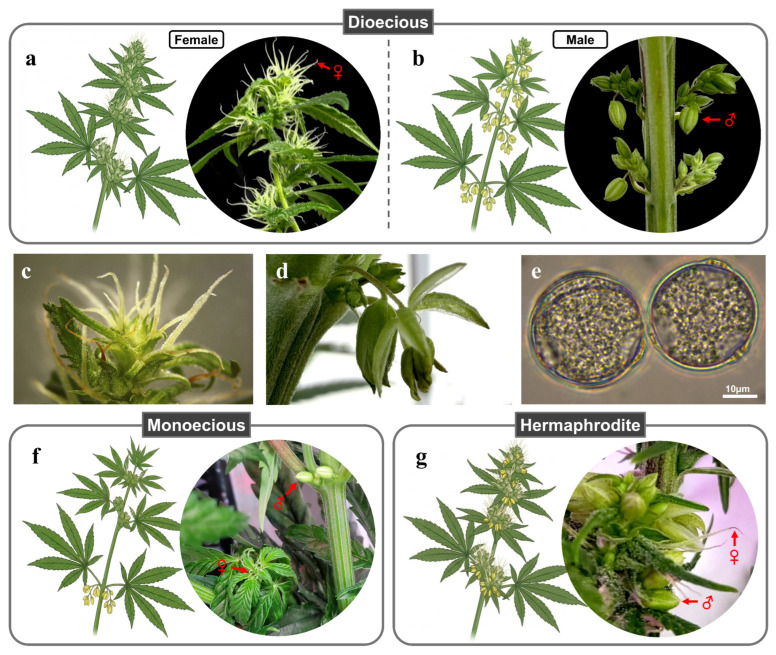
Sexual systems and floral organization in *Cannabis sativa* L. (**a**,**b**) Dioecious plants showing separate female (**a**) and male (**b**) individuals. In dioecious *C. sativa*, female and male plants develop pistillate female flowers (**c**) and staminate male flowers (**d**), respectively, on separate individuals. (**e**) Micrograph of pollen produced by a male flower. (**f**) Monoecious plant bearing both female and male flowers at distinct spatial locations, with pistillate flowers typically formed at apical regions and staminate flowers arising along the stem. (**g**) Hermaphroditic expression characterized by the simultaneous presence of female and male reproductive structures within the same inflorescence, often with anthers developing within otherwise pistillate floral tissues. The red arrows indicate the female (♀) and male (♂) reproductive organs. The graphics were created using BioRender (biorender.com).

*C. sativa* belongs to the family Cannabaceae and is primarily a dioecious plant, in which female and male individuals are typically separate (Figure 1a,b). Dioecy, while relatively rare among angiosperms (~5 to 6% of species), is phylogenetically widespread and has evolved repeatedly across flowering plant lineages [5,6,7,8]. *C. sativa* is known as a wind-pollinated plant and typically reproduces through the pollination of female flowers (Figure 1c) by pollen dispersed from mature male flowers (Figure 1d,e) [9]. As an evolutionary strategy, dioecy promotes obligate outcrossing, enhancing genetic diversity and population fitness. However, dioecious systems also require strict control of pollen dynamics in cultivation settings, particularly in cannabinoid-focused production systems where fertilization reduces phytocannabinoid accumulation, alters terpenoid composition, and produces seeded inflorescences that are undesirable for smokeable floral products [3,10].

In addition to dioecy, monoecious phenotypes are observed in *C. sativa* germplasm and can be maintained through breeding [10,11]. Monoecy refers to the presence of spatially separated male and female flowers on the same individual plant (Figure 1f). In cannabis, female flowers are often more prevalent in the upper (apical) regions, which may favor exposure to airborne pollen, whereas male flowers tend to occur along the stem or in lower (basal) regions, where their position may support pollen shedding and dispersal. However, this distribution is not strictly maintained, and spatial arrangement can vary with genotype and environmental conditions [12,13].

Monoecy represents a stable reproductive condition that has been widely exploited in hemp production to reduce crop heterogeneity, facilitate synchronized development, and enable dual-purpose harvesting of fiber and seed [2]. Despite these advantages, monoecious populations may retain residual variability in sex expression, including variation in sex ratios and the occurrence of unisexual or masculinized individuals, and therefore require continued selection to maintain stability, underscoring the dynamic nature of reproductive traits in *C. sativa* [11,13,14].

In contrast to these stable systems, mixed-sex phenotypes are also present and reflect plasticity in sex expression. Among these, hermaphroditism is the most prominent form, characterized by the co-occurrence of female and male flowers at the same spatial location (Figure 1g), and is typically associated with instability in reproductive development [10]. Such phenotypes may arise spontaneously or in response to environmental or physiological factors and have important implications for both plant reproduction and crop management [10,15]. Under conditions of pollen limitation or environmental stress, shifts toward hermaphroditic or mixed-sex expression may enhance reproductive assurance by facilitating self-fertilization when outcross pollen is limited, although increased selfing may reduce genetic diversity [6,10,16].

In commercial drug-type cannabis, spontaneous hermaphroditic inflorescence has been documented in ‘Moby Dyck’, ‘Space Queen’, and ‘Lemon Nigerian’, with anthers appearing within pistillate inflorescences in approximately 5–10% of examined plants. However, the physiological and environmental factors underlying this response remain poorly understood [10]. Although QTL and cytogenetic studies indicate a genetic basis for variation in sex-expression in cannabis, current evidence has not identified specific cannabinoid cultivars with a confirmed predisposition to spontaneous hermaphroditism [12,13,17].

Given the complexity and variability of sex expression in *C. sativa*, this review examines the genetic and regulatory basis of sex determination and plasticity in *C. sativa*, with a particular focus on hermaphroditic expression and its implications for crop performance. In addition to genetic regulation, we further examine the influence of environmental factors, as well as exogenous hormones and chemical treatments, on shifts in sexual phenotype. Considering the significant impact of unintended pollination on cannabinoid yield and flower quality by introducing seed set in intended sinsemilla crop, we highlight the need for reliable early detection and review emerging approaches for identifying hermaphroditic traits. Finally, we discuss the commercial implications of sex instability and identify key knowledge gaps that must be addressed to improve the predictability and management of sex expression in *C. sativa*.

## 2. Genomic Architecture and Regulation of Sex Determination in *Cannabis sativa* L.

Sex expression in *C. sativa* is governed by a complex interplay of chromosomal, hormonal, and polygenic regulatory mechanisms and is not solely determined by sex chromosomes. Although the species is diploid (2n = 20), comprising nine pairs of autosomes and one pair of sex chromosomes, accumulating evidence indicates that chromosomal composition alone is insufficient to explain sex determination [17,18,19]. Consistent with observations in hop, cytogenetic studies support an X-to-autosome (X:A) balance component, whereby sex expression is influenced by the relative dosage of X-linked and autosomal factors and can be modulated by environmental conditions or plant growth regulators despite stable chromosomal sex [17].

Early models of sex determination proposed either a single-locus epistatic switch, in which a dominant male-determining region overrides the default developmental pathway, or a two-locus system comprising a male-determining gene (M) and a suppressor of female development (SuF) [5,20,21]. However, recent genomic and pangenomic analyses refine this framework, supporting a dosage- and pathway-dependent model in which sex-linked regions interact with autosomal loci and hormonal signaling pathways [15,22]. In this context, sex-biased genes are widely distributed across autosomes and pseudoautosomal regions, and quantitative trait locus (QTL) analyses have identified multiple loci associated with sex expression, including genes involved in flowering time, photoperiod sensitivity, and hormone signaling [12,13]. This distributed architecture provides a mechanistic basis for the plasticity of sex expression and the emergence of alternative phenotypes under environmental or physiological perturbations [10].

Recent pangenome-scale analyses further demonstrate that *C. sativa* possesses an ancient heteromorphic XY system, with sex chromosomes originating more than 36 million years ago, placing cannabis among the oldest known plant systems with differentiated sex chromosomes [23]. Haplotype-resolved assemblies reveal substantial structural divergence between the X and Y chromosomes, with the Y chromosome (~108–113 Mb) larger than the X (~83–85 Mb) due to repeat expansion and the formation of a large non-recombining sex-determining region (SDR; ~79–84 Mb) [22,23]. Patterns of synonymous divergence indicate that the SDR expanded progressively from the pseudoautosomal boundary toward the centromere, consistent with stepwise recombination suppression driven by both selection and neutral processes [23].

Importantly, gene content is not confined to the SDR. The pseudoautosomal region retains higher gene density and includes key regulators of flowering and developmental timing, such as *FLOWERING LOCUS T*, *CONSTANS*, and *GIGANTEA*, while male-biased genes are distributed across the SDR, pseudoautosomal regions, and autosomes [23]. These findings reinforce a distributed, network-based model of sex regulation rather than a strictly chromosome-limited system. Consistent with this framework, sex determination in *C. sativa* is not governed by a canonical Y-linked master regulator. Instead, key regulatory loci are associated with the X chromosome and autosomes, particularly those involved in ethylene biosynthesis and signaling, including aminocyclopropane-1-carboxylate synthase (ACS), which influences floral sex differentiation [22,24].

Within this genomic context, sexual phenotypes can be viewed as a continuum. Chromosomal sex (XX/XY) generally underlies dioecious expression, but monoecious individuals (typically XX) demonstrate that both female and male floral programs can be expressed within a single genetic background through stable modulation of regulatory pathways [17,22].

Collectively, these findings support a multilayered, dosage-sensitive, and pathway-dependent model of sex determination in *C. sativa*. These features of integrating chromosomal structure, autosomal modifiers, hormonal signaling, and environmental regulation, together with exogenously inducible hermaphroditic expression [10,24], support the mechanistic basis underlying the plasticity of sex expression observed in *C. sativa*.

## 3. Agronomic and Commercial Implications of Dioecious, Monoecious, and Hermaphroditic Systems in *Cannabis sativa* L.

Dioecious systems in *C. sativa* are central to modern production systems, with their agronomic value determined largely by sex-specific functional specialization. Female (pistillate) plants are the primary target in drug-type cultivation due to their high accumulation of cannabinoids in unpollinated inflorescences, which contain dense glandular trichomes [10]. Maintaining sinsemilla (unseeded female crop) conditions is therefore essential for maximizing cannabinoid yield and product quality. In addition to their chemical value, pistillate plants may also exhibit enhanced resistance to herbivores and pathogens [25,26].

However, this system is highly sensitive to unintended pollination. Pollen dispersal within cultivation environments can result in fertilization that reduces Δ^9^-tetrahydrocannabinolic acid (Δ^9^-THCA) and cannabidiolic acid (CBDA) content by approximately 60–75% [3]. Importantly, this risk extends beyond male plants, as hermaphroditic flowers also produce viable pollen capable of achieving seed set, with seed viability rates reaching 90–95% [10]. Consequently, the unintended emergence of pollen-producing individuals represents a major constraint in cannabinoid-focused cultivation, as it can trigger widespread pollination, reduce potency, and result in seeded inflorescences unsuitable for floral markets.

In contrast, male (staminate) plants, while undesirable in cannabinoid-focused systems, play an important role in fiber and seed production. In fiber-type cultivars, male plants often exhibit traits favorable for industrial applications, including rapid growth, increased height, shorter life cycles, and the production of finer fibers suitable for textile use [27,28]. In breeding and seed production systems, staminate plants are also essential for controlled pollination. However, dioecious cultivation introduces management challenges due to differences in reproductive timing between sexes [28,29]. Staminate plants typically flower and senesce earlier than pistillate plants, requiring precise coordination for pollen collection and seed production [29]. Field studies further highlight this temporal variability, with male flowering preceding female flowering by approximately 20 days in Carmagnola and by 15–60 days in Fibranova depending on sowing date, while flowering duration across hemp genotypes ranges from 4 to 99 days. Such variability complicates crop synchronization and the identification of a single optimal harvest window [29,30].

In light of these limitations, monoecious systems offer an alternative strategy that integrates reproductive functions within a single plant and is widely utilized in industrial hemp production. These systems are particularly advantageous for reducing crop heterogeneity and facilitating uniform growth and maturation, thereby improving management efficiency [28,29]. Their suitability for dual-purpose production is well established, with monoecious cultivars supporting simultaneous stem and seed yields under optimized conditions, including approximately 12.5 t ha^−1^ stem and 1.9 t ha^−1^ seed under mid-April sowing in early or mid-early cultivars such as Fedora 17 and Felina 32 [29,30]. In addition, the monoecious system simplifies cultivation practices by eliminating the need for separate staminate plants and reducing the risk of unintended pollen contamination within controlled genetic backgrounds [29,31].

Despite these advantages, monoecious populations are not entirely stable under field conditions. Residual variability in sex expression, including shifts in sex ratios and the occurrence of unisexual or masculinized individuals, can lead to gradual reversion toward dioecy without continued selection [11,12,13,14]. Thus, while monoecy enhances uniformity and management efficiency, its long-term stability requires sustained breeding efforts.

In this context, hermaphroditism represents a distinct and often problematic manifestation of sex expression plasticity in *C. sativa*. Although only occasionally observed, hermaphroditic expression in female plants can have disproportionate consequences in commercial production systems. The spontaneous development of male reproductive structures within pistillate inflorescences can result in internal pollen release, leading to unintended seed set and reductions in floral quality and cannabinoid yield [10]. Such events are often associated with environmental and operational stressors, including disruption of the dark period, temperature fluctuations, and irregularities in lighting systems in controlled cultivation environments [10,32]. These factors have been implicated in hermaphroditic or staminate development in female *C. sativa*, and the resulting self-pollination or unintended fertilization can reduce floral quality and phytocannabinoid accumulation while contributing to economic losses in commercial production systems [3,9].

Despite these challenges, hermaphroditism can be strategically exploited in breeding programs. Induced hermaphroditic plants can produce pollen lacking a Y chromosome and are therefore widely used to generate feminized seeds [33]. This approach enables the production of predominantly female progeny, supporting consistent cannabinoid yield and reducing the need for removal of male plants in cultivation systems.

Collectively, these reproductive systems reflect trade-offs between specialization, uniformity, and stability of sex expression in *C. sativa*. Dioecious systems enable optimization of sex-specific traits but require strict control of pollination and reproductive timing. Monoecious systems improve uniformity and support efficient dual-purpose production, although their stability may decline without continued selection. In contrast, hermaphroditism represents a problematic manifestation of sex expression, as unintended pollen production can lead to seed set and reduced cannabinoid yield. Despite its utility in feminized seed production, it remains a key constraint in commercial cultivation.

## 4. Hermaphroditic Expression in *Cannabis sativa* L.

Given that sex expression in *C. sativa* is not strictly determined by chromosomal constitution, floral development remains highly responsive to internal hormonal balance and external influences. At the physiological level, shifts in endogenous hormone signaling, particularly involving ethylene and gibberellins, play a central role in destabilizing sex expression and predisposing plants to hermaphroditic or mixed sex development.

Building on this hormonal plasticity, exogenous chemical treatments can further perturb sex determination pathways. Application of plant growth regulators or hormone-modulating compounds has been shown to induce alterations in sexual phenotype in both monoecious and dioecious forms, reflecting the sensitivity of floral development to externally imposed hormonal cues [12,34].

Environmental factors act as additional modulators of this system. Variations in photoperiod, temperature, water availability, and nutrient status have all been associated with increased incidence of sex instability. These abiotic stressors may act independently or synergistically with hormonal and chemical inputs, amplifying disruptions in developmental regulation [11]. Within this integrated framework of hormonal control, chemical perturbation, and environmental responsiveness, hermaphroditic expression in *C. sativa* emerges as a consequence of the dynamic interplay among these factors.

### 4.1. Hormonal Regulation of Sex Expression

Sex expression in *C. sativa* is strongly regulated by endogenous phytohormones and their interactions. Across both monoecious and dioecious plant species, ethylene, auxin, cytokinins, and abscisic acid (ABA) are generally associated with promotion of female flower development, whereas gibberellins (GAs) promote male flower formation [35,36,37].

Ethylene (C_2_H_4_) is synthesized from methionine through ACC intermediates via ACC synthase (ACS) and ACC oxidase (ACO). Auxin biosynthesis produces indole-3-acetic acid (IAA) primarily through tryptophan-dependent YUCCA pathways. Cytokinins such as trans-zeatin are generated through isopentenyltransferase (IPT)- and LONELY GUY (LOG)-mediated pathways, whereas ABA is synthesized through carotenoid cleavage involving 9-cis-epoxycarotenoid dioxygenase (NCED). Bioactive gibberellins, including GA_1_ and GA_4_, are synthesized through the terpenoid pathway via GA20ox- and GA3ox-mediated reactions [38].

Rather than the biosynthetic pathways themselves, the endogenous balance, spatial distribution, and signaling activity of these hormones appear to be the primary determinants of floral sex differentiation and sexual plasticity in cannabis. Ethylene has been particularly implicated as a key feminizing hormone in *C. sativa*, whereas GA accumulation is closely associated with staminate flower formation and male sex expression [11,39]. Auxin and cytokinin signaling are also linked with female floral development, likely through regulation of meristem activity, floral organ differentiation, and ethylene-responsive pathways. ABA, although primarily associated with abiotic stress responses, may additionally contribute to stress-mediated sexual plasticity in cannabis.

Importantly, accumulating evidence suggests that sex expression in *C. sativa* is highly genotype dependent, with substantial variation in hormone biosynthesis, signaling sensitivity, and downstream transcriptional responses among cultivars [40]. Ethylene-associated genes, including *ACS1*, *ACO5*, *ERF1*, and *MTN*, have shown cultivar-dependent expression patterns during cannabis sexual plasticity, further supporting the central role of ethylene signaling in feminization and floral sex determination [41]. Likewise, differential expression of auxin-related genes including *IAA-1*, *IAA-2*, *X15-1*, and *X15-2*, as well as the ABA-associated gene *PP2C-1*, has been reported among cannabis genotypes [42]. Given the extensive genomic diversity of *C. sativa* germplasm [23], these findings collectively suggest that endogenous hormone regulation and genotype-dependent hormonal responsiveness are major determinants of floral development and sex expression in cannabis.

#### 4.1.1. Ethylene-Mediated Feminization

Based on hormone-mediated regulation of sex-expression, exogenous hormones or hormone-modulating compounds can be utilized to manipulate floral sex for breeding, feminized seed production, and cultivar stabilization in *C. sativa* [43].

Among these pathways, ethylene plays a central role in promoting pistillate flower development [44,45]. Consistent with its feminizing role, exogenous application of ethylene-releasing compounds has been shown to induce female flower formation even in genetically male plants, further supporting ethylene as a key regulator of cannabis sex determination and sexual plasticity [45,46].

Ethephon, an ethylene-releasing compound, has been widely used to induce feminization in *C. sativa*. In the dioecious Thai hemp cultivar RPF3, ethephon application (250–1000 ppm) during early male bud development resulted in complete morphological feminization and the formation of functional seed-bearing female inflorescences in genetically male (XY) plants [46]. These findings suggest that male floral fate in cannabis remains developmentally reversible during early stages of floral differentiation.

However, feminization induced by ethephon is not always complete, and treated plants may produce mixed-sex inflorescences, indicating partial sexual plasticity. Transcriptomic analyses further suggest that ethylene-mediated feminization extends beyond phenotypic changes and involves reprogramming of floral organ identity pathways. In particular, altered expression of class B and E floral homeotic genes, major components of the ABCDE floral development model responsible for stamen and floral organ specifications, indicates that ethylene signaling directly influences developmental programs governing unisexual flower formation in *C. sativa* [47].

**Figure 2:**
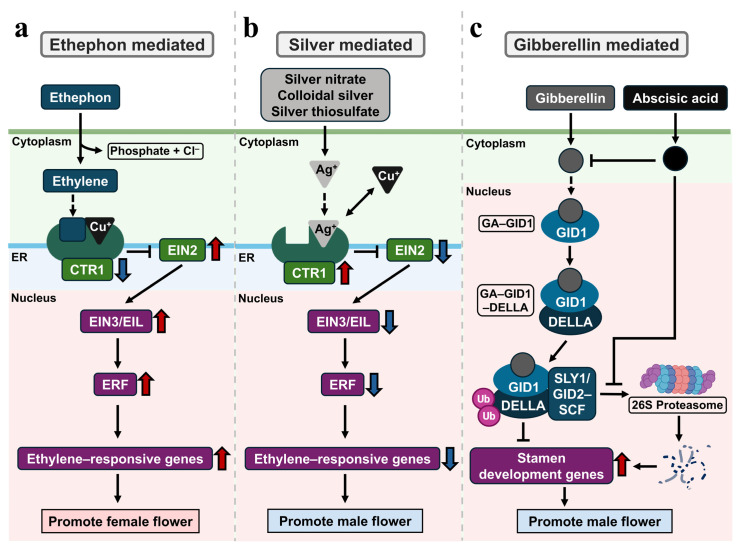
Potential mechanisms of exogenous hormonal regulators altering sex expression in *Cannabis sativa*. Red upward arrows indicate overexpression or activation, whereas blue downward arrows indicate downregulation or inactivation. Black arrows denote the sequential progression of the pathway, blunt-ended lines represent antagonistic interactions, and dashed arrows indicate receptor binding. (**a**): Ethephon releases ethylene and activates ethylene-responsive signaling, thereby promoting female flower development. (**b**): Silver-based compounds inhibit ethylene perception through Ag^+^, suppressing ethylene signaling and promoting male flower formation. (**c**): Gibberellins (GAs) promote male flower formation through GA signaling, whereas ABA antagonizes this response.

Ethylene signaling is initiated by ethylene perception through receptor complexes localized on the endoplasmic reticulum membrane (Figure 2a) [48]. Ethylene receptors such as ETR1 require a copper cofactor for proper ethylene binding and signal perception [49]. In the absence of ethylene, these receptors activate the downstream negative regulator CTR1, which suppresses EIN2 activity. Upon ethylene binding, receptor and CTR1 kinase activities are inhibited, thereby relieving CTR1-mediated repression of EIN2 [50]. Activated EIN2 subsequently promotes stabilization and activation of nuclear EIN3/EIL transcription factors, which induce the expression of ethylene response factor (ERF) genes and other ethylene-responsive targets [51]. This transcriptional cascade is proposed to positively regulate downstream developmental pathways associated with female flower formation and sexual plasticity in *C. sativa* [52].

Studies in cucumber (*Cucumis sativus*) further support the role of ethylene-responsive transcriptional networks in sex determination. Pan et al. (2021) demonstrated that CsERF31 promotes female flower differentiation through activation of *CsACS2*, thereby reinforcing ethylene biosynthesis via a positive feedback loop [53]. Similar regulatory mechanisms may contribute to ethylene-mediated feminization and sexual plasticity in *C. sativa*, although additional functional studies are needed to clarify these pathways in cannabis.

#### 4.1.2. Silver-Based Ethylene Inhibitors

In contrast to the ethephon-mediated feminization, masculinization of sex expression in *C. sativa* is most effectively achieved through inhibition of ethylene signaling [39]. Silver-based compounds, including silver nitrate (AgNO_3_), colloidal silver, and silver thiosulfate (STS), function as anti-ethylene agents by delivering Ag^+^ ions that interfere with ethylene perception at the receptor level (Figure 2b) [54,55]. Ethylene receptors such as ETR1 require a copper cofactor for proper ethylene binding, and Ag^+^ ions disrupt this metal-dependent receptor system, thereby inhibiting downstream ethylene signal transduction [55]. As a consequence, ethylene-responsive feminization pathways are suppressed, promoting staminate flower development and male sex expression in genetically female plants (Figure 2b) [54].

Among these treatments, silver nitrate was one of the earliest compounds used for cannabis sex reversal. Application of AgNO_3_ to genetically female plants induced male and intersexual flowers capable of producing viable pollen and seeds. However, its practical use has been limited by phytotoxicity, inconsistent efficacy, and narrow dose tolerance [56]. Colloidal silver subsequently emerged as a more accessible alternative and has been widely used in commercial cannabis production. Although colloidal silver can induce viable male flowers and pollen production in female plants, its effectiveness often varies depending on genotype, concentration, and treatment regime [33].

Currently, STS is regarded as the most reliable chemical masculinization agent in cannabis due to its enhanced stability, tissue mobility, and stronger inhibition of ethylene signaling [57]. Early studies demonstrated that STS induced significantly greater numbers of fully developed male flowers than silver nitrate, with resulting pollen capable of successful fertilization and seed production [56]. Lubell and Brand further demonstrated that three STS applications at 7-day intervals during short-day induction produced near-complete masculinization in female hemp plants, with terminal inflorescences achieving greater than 95% male flower conversion at 3 mM STS. [58]. More recent studies confirm that optimized STS concentrations ranging from 0.3 to 1.5 mM effectively induced male flowers with high pollen viability across multiple modern hemp cultivars [59].

Nevertheless, Wizenberg et al. reported that unisexual male plants produced approximately 223% more pollen than STS-induced cosexual plants [60]. Similarly, Lubell and Brand observed that pollen release from chemically induced male flowers was less efficient than that of naturally occurring male plants [58]. In addition, Kim et al. (2024) demonstrated that the effectiveness of STS treatment is highly dependent on application timing, treatment frequency, and cultivar background [59]. Therefore, although STS is highly effective for inducing masculinization, successful application requires careful optimization of concentration, timing, and treatment regime to achieve consistent pollen production and reliable sex-reversal outcomes.

Overall, inhibition of ethylene signaling using silver-based compounds represents the most reliable strategy for inducing masculinization in modern cannabis germplasm. However, silver-based treatments also raise environmental and toxicological concerns. Because these compounds rely on Ag^+^ ions, residual silver may persist in waste solutions [61,62], plant tissues [62], or cultivation substrates following application [62]. Therefore, although STS and related silver-based compounds remain highly effective tools for induced masculinization, their use requires careful handling, waste management, and disposal practices to minimize unintended environmental contamination.

#### 4.1.3. Gibberellin-Mediated Masculinization

Gibberellins (GAs) act as primary masculinizing agents by redirecting floral meristems toward staminate differentiation. Early studies by Ram and Jaiswal (1972) demonstrated that exogenous application of gibberellins, including GA_3_, GA_4+7_, GA_7_, and GA_9_, induced male flower formation in genetically female plants [63]. Repeated application to shoot apices over a 10-day period resulted in male flower development at newly formed nodes. At 50 μg GA_3_ per plant, treated individuals produced an average of 3.6 male-bearing nodes and approximately 33.8 male flowers per plant, with floral conversion occurring primarily on the third through sixth newly formed nodes 2–3 weeks after treatment [63].

More recently, Garcia-de Heer et al. (2025) further confirmed the masculinizing role of GA_3_ by demonstrating female-to-male sex reversion in genetically female *C. sativa* plants [47]. Notably, combined treatment with GA_3_ and STS produced extensive masculinization, with modeled probabilities of male flower production reaching 86% and 99% in the Romanian and Syrian female lines, respectively [47]. Importantly, both studies demonstrated that GA-associated masculinization produced functionally male reproductive structures, as the induced staminate flowers developed morphologically normal stamens and viable pollen. These findings suggest that GA treatment can initiate a complete staminate developmental program rather than merely inducing aberrant floral morphology.

Gibberellin signaling is initiated through perception of bioactive GAs by the GA-Insensitive Dwarf1 (GID1) receptor located in the nucleus and cytoplasm [64]. When GA binds to GID1, the receptor undergoes a conformational change that enables it to interact with DELLA proteins, the key repressors of GA responses, resulting in the formation of a GA-GID1-DELLA complex (Figure 2c) [65] Upon formation of this GA-GID1-DELLA complex, DELLA proteins undergo a structural change that allows them to be recognized by specific F-box proteins, such as SLY1 in *Arabidopsis thaliana* or GID2 in rice [66,67]. These F-box proteins confer E3 ubiquitin ligase activity on the SCF (SKP1-CUL1-F-box) complex toward DELLA, enabling ubiquitination of DELLA proteins. The SCF complex successively attaches ubiquitin molecules to the targeted DELLA proteins, generating a degradation signal that leads to DELLA proteolysis by the 26S proteasome (Figure 2c) [66].

In *A. thaliana*, DELLA proteins suppress expression of floral organ identity genes associated with stamen development, including *AP3*, *PI*, and *AG* [68]. Similarly, suppression of the DELLA-family gene *SpGAI* in spinach (*Spinacia oleracea*) has been shown to induce masculinization of female flowers [69]. These findings collectively suggest that GA-mediated masculinization is primarily achieved through DELLA degradation and subsequent activation of stamen identity pathways.

In contrast, ABA acts antagonistically to GA-induced masculinization, suppressing GA-mediated male flower formation. ABA alone failed to induce male flowers, and application of 50–100 μg ABA per plant completely suppressed GA_3_-induced masculinization, while higher GA_3_ concentrations only partially overcame this inhibition [63]. This antagonistic effect can be interpreted at the metabolic level as ABA suppressing the expression of GA biosynthetic genes, such as members of the *GA3ox* and *GA20ox* families, thereby reducing GA accumulation [70]. Furthermore, ABA is known to reduce the availability of bioactive GA and increase DELLA stability, thereby decreasing DELLA ubiquitination and proteasome-mediated degradation (Figure 2) [71]. Therefore, this ABA-mediated mechanism may suppress GA-mediated masculinization by maintaining DELLA-dependent repression of GA-responsive transcriptional programs involved in stamen development and staminate flower formation [70,72].

### 4.2. Environmental Stressors and Sex Expression Stability

Environmental stressors have long been considered potential contributors to sex-expression instability in *C. sativa*, particularly in relation to hermaphroditic or intersexual flower formation.

Photoperiod disruption has frequently been associated with altered sex expression in cannabis. Although Oliver et al. (2024) reported no significant association between low-intensity light leakage during the dark period and male flower formation [32], an earlier study by Borthwick and Scully (1954) demonstrated that approximately 25–45% of female plants produced male flowers under 8- and 11 h photoperiods, while male flower formation was suppressed under a 14 h photoperiod [73]. These findings support a relationship between photoperiodic conditions and sex-expression stability in *C. sativa*, while also indicating that the outcome may depend on light intensity, exposure duration, developmental stage, and the specific photoperiodic context.

Temperature stress has also been associated with altered floral sex expression. In cucumber, high temperature (day/night, 32 °C/24 °C) and long-day photoperiod (day/night, 16 h/8 h) suppressed female flower formation across a large germplasm set, with 71.3% of 359 accessions showing significantly reduced femaleness in early autumn compared with spring [74]. Transcriptomic and epigenetic analyses linked these responses to phytohormone-associated pathways, floral development genes, and DNA methylation changes involving *AGAMOUS*, *CAULIFLOWER A*, and *CsACO3* [74]. Although these mechanisms have not yet been confirmed in cannabis, they support the hypothesis that epigenetic regulation and floral organ identity pathways may contribute to environmentally induced sexual plasticity in *C. sativa*.

Low temperature has also been reported to promote male flower formation in female hemp plants. Borthwick and Scully (1954) showed that exposure to approximately 13 °C before photoperiodic induction increased the proportion of female plants producing male flowers by approximately 26 percentage points compared with plants exposed to approximately 21 °C [73]. Although the molecular mechanisms underlying these photoperiod- and temperature-associated responses have not been resolved in *C. sativa*, comparative studies in other sexually plastic species provide useful mechanistic context. Temperature fluctuation should also be considered separately from mean temperature. In photo-thermo-sensitive male-sterile wheat, a 15 °C daily temperature difference induced complete male sterility and was associated with transcriptomic changes in MAPK signaling, starch and sucrose metabolism, phenylpropanoid biosynthesis, flavonoid biosynthesis, and cutin, suberine, and wax biosynthesis [75]. This is relevant to controlled-environment cannabis production because canopy microclimates, HVAC cycling, and day-night temperature differentials may impose fluctuating stress conditions that are not captured by mean temperature alone.

Reproductive-stage sensitivity to abiotic stress has also been documented in cereal systems. In wheat, combined heat and drought stress during gametogenesis reduced pollen viability, altered pistil morphology and anatomy, increased ROS and RNS generation, intensified lipid peroxidation, and decreased nitric oxide production in stigmatic papilla cells [76]. Although these studies do not directly model cannabis hermaphroditism, they highlight the importance of evaluating reproductive tissue integrity, pollen viability, oxidative stress, and floral development alongside visible sex-expression phenotypes in cannabis stress studies.

Nutrient imbalance has also been proposed as a potential contributor to sex-expression instability in *C. sativa*, although controlled studies establishing specific nutrient thresholds or fertilizer regimes associated with hermaphroditic flower formation remain limited.

Collectively, current evidence suggests that environmental stressors may contribute to sex instability in *C. sativa* [11,12,73]. Future studies should therefore use factorial designs that separate cultivar, photoperiod continuity, mean temperature, daily temperature fluctuation, water availability, nutrient status, and light intensity or spectrum [74,75,76]. Such studies would allow environmental stressors to be evaluated as genotype-dependent risk factors rather than as universal or deterministic causes of hermaphroditic expression.

### 4.3. Factors Affecting Sex Reversal Efficiency

Sex expression in *C. sativa* is strongly influenced by cultivar-specific genetic background, treatment regime, and the developmental stage at which induction is applied. Responses to identical treatments vary substantially among cultivars. For example, Lubell and Brand (2018) found most genotypes show only partial masculinization at 0.3 mM STS, such as their industrial hemp achieving 42% conversion whereas CBD hemp A achieved up to 91% under the same conditions, indicating pronounced genotype-dependent sensitivity [58]. Similarly, variation in both sex-reversal efficiency and the rate of male flower development has been reported across cannabis varieties [15].

In addition to genetic variation, treatment regime further modulates these responses by influencing the magnitude and consistency of hormonal disruption. Differences in compound type, concentration, and application regime can lead to variable outcomes even within the same genotype. For example, repeated STS applications produce more reliable and near-complete masculinization compared with lower or single-dose treatments, whereas alternative compounds such as AVG, cobalt nitrate, and 1-MCP exhibit reduced efficacy or undesirable side effects [58,77].

Beyond genotype and treatment conditions, developmental stage imposes a critical constraint by defining the window during which floral tissues remain responsive to hormonal reprogramming. Reproductive commitment has been shown to occur as early as the fourth node, indicating that successful induction depends on treatment prior to fixation of floral organ identity [11]. Consistent with this, multi-omics analyses demonstrate that ethylene-mediated sex reprogramming is initiated before visible floral differentiation, with transcriptional responses detectable within approximately 18 h of treatment and subsequently diverging into stable XX and XY developmental trajectories during phenotype stabilization [54].

Taken together, these observations support a unified model in which the efficiency of chemically induced sex reversal is determined by cultivar-specific differences in baseline hormone balance, sensitivity to ethylene inhibition, and the developmental window of floral responsiveness. This phase-dependent regulation highlights that sex determination in *C. sativa* is governed by coordinated, context-dependent processes rather than a single binary switch.

## 5. Detection and Early Identification of Sex Expression Instability

Because hermaphroditic conversion can occur after crop establishment and before obvious yield loss becomes visible, early detection is essential in commercial *C. sativa* production. Fertilization in crops intended to remain sinsemilla reduces phytocannabinoid accumulation, alters terpenoid composition, and initiates unwanted seed formation, with direct consequences for floral quality, harvest value, and pollen-mediated spread to adjacent plants. Continuous monitoring is therefore necessary in production systems where developmental instability or cultivation stress may promote anther formation [10,78].

Visual inspection remains the primary detection method, but its effectiveness is limited by the morphology and location of hermaphroditic structures. In *C. sativa* inflorescences, individual anthers or small clusters of anthers typically arise within bract tissues adjacent to the stigmas and may first become visible during weeks 4 to 7 of flowering. These yellow, 2–3 mm structures often emerge within otherwise pistillate flowers, and in more severe cases, entire pistillate flowers may convert to staminate forms [10].

Because such structures can be sparse, localized, and partially concealed, early identification during routine scouting is difficult. This limitation is particularly significant because hermaphroditic anthers are functional: Punja and Holmes (2020) reported that hermaphroditic flowers produced mature seeds before harvest, and those seeds germinated at rates of 90–95% within 10–14 days. Detection must therefore occur before pollen release and fertilization rather than after visible seed set or extensive floral conversion has already occurred [10].

### 5.1. Raman Spectroscopy

Where visual inspection is insufficient, spectroscopic approaches may provide earlier and more objective discrimination. Earlier handheld Raman studies showed that fresh plant material could be differentiated between CBDA- and THCA-dominant cannabis types [79]. Building on this foundation, Raman spectroscopy has emerged as a promising noninvasive tool for distinguishing female, male, and hermaphroditic plants.

Goff et al. (2022) [78] showed that Raman spectra from these three plant classes differed consistently in carotenoid-associated bands at 1156, 1186, and 1218 cm^−1^, which could serve as quantitative marker bands for discrimination. Hermaphroditic plants also displayed a distinct Amide I signal centered near 1650 cm^−1^, whereas the corresponding feature in female and male plants was shifted to approximately 1680 cm^−1^, indicating additional differences in protein-associated spectral structure.

Using a handheld Raman spectrometer coupled with machine-learning analysis, the authors achieved 98.7% accuracy for hermaphrodite identification and 100% accuracy for female and male plants. These findings indicate that Raman spectroscopy may serve as a practical confirmatory tool when visual inspection is equivocal or when high-throughput, nondestructive screening is needed in commercial production systems [78]. Although Raman spectroscopy shows strong potential for nondestructive identification of hermaphroditic plants, several limitations should be considered. Classification models are dependent on the dataset used for training and may be influenced by cultivar background, sampled tissue, developmental stage, and production conditions. Spectral quality may also vary with instrument configuration, laser wavelength, sample pigmentation, fluorescence background, and preprocessing method. In addition, handheld Raman systems remain relatively costly, which may limit routine use in some cultivation settings [78,80]. Therefore, Raman spectroscopy remains a promising screening tool, but broader validation is still needed before routine commercial use.

### 5.2. Sex-Linked Markers and QTLs Relevant to Hermaphroditism in Cannabis sativa *L.*

Phenotypic screening can be complemented by molecular approaches that identify sex-linked variation and loci associated with sex-expression instability before overt floral conversion becomes visible. Sex-linked molecular markers in *C. sativa* were first identified in the 1990s using random amplified polymorphic DNA methods. The first male-associated marker (Male-Associated DNA from Cannabis-1; MADC1) did not contain an open reading frame but was later shown to include long interspersed nuclear element-like sequences concentrated on the short arm of the Y chromosome [81,82] (Table 1).

Subsequent work identified MADC2, a 390 bp marker developed using sequence-characterized amplified region methods that reliably distinguishes males across numerous hemp cultivars [83] (Table 1). Later studies also identified MADC3 and MADC4, which correspond to copia-like retrotransposons and produce strong fluorescent in situ hybridization (FISH) signals on the Y chromosome [84]. More recent molecular assays further support the utility of early sex identification for distinguishing genetic female and male plants, but these approaches do not capture phenotypic instability and therefore cannot detect hermaphroditism in otherwise female plants [85].

**Table 1:** Sex-identification markers in *Cannabis sativa* L.

Sex Association	Locus Name	Locus Description	Primer Name	Primer Sequence (5′ → 3′)	Annealing Temp. ^(1)^	Amplicon Size	Reference
Male	CSP-1	SNP ^(2)^ region of MADC ^(3)^ 6	CSP1-FAM	F: GAAGGTGACCAAGTTCATGCTA	-	-	[86]
				R: GCTTGAAATGAGATGTCAAACC			
			CSP1-HEX	F: GAAGGTCGGAGTCAACGGATTG	-	-	
				R: AGCTTGAAATGAGATGTCAAACT			
	MADC1	RAPD ^(4)^ (No. 11) base male specific region	No. 11	ACGGCATATG	34 °C	730 bp	[81]
	MADC2	RAPD (OPA8) base male specific region	MADC2	F: GTGACGTAGGTAGAGTTGAA	60 °C	390 bp	[17,83]
				R: GTGACGTAGGCTATGAGAG			
	MADC3	RAPD (OPB-18) base male specific region	OPB-18	CCACAGCAGT	34 °C	771 bp	[84]
	MADC4	RAPD (OPC-04) base male specific region	OPC-04	CCGCATCTAC	34 °C	576 bp	
Female	-	-	OPA-04	AATCGGGCTG	36 °C	870 bp	[87]
	-	-	OPF-05	CCGAATTCCC	36 °C	1160 bp	

Blank (-) cells indicate that the corresponding information was not reported in the original publication. ^(1)^ Temp.: Temperature, ^(2)^ SNP: Single Nucleotide Polymorphism, ^(3)^ MADC: Male-associated DNA from cannabis, ^(4)^ RAPD: Random Amplified Polymorphic DNA.

Later markers such as MADC5 and MADC6 were found in both sexes, which reduced their diagnostic value and prompted the development of improved systems such as *Cannabis sativa* L. Subtelomeric Repeat Probe (CSP-1) [86]. Amplified fragment length polymorphism studies additionally revealed recombination between the X chromosome and the pseudoautosomal region of the Y chromosome and identified shared sex-linked fragments in both dioecious and monoecious populations [12]. Female-specific markers such as OPA-04 and OPA-05 [87] and the CSP-1-FAM system [86] appear to result from gene deletions or epigenetic regulation affecting male genomes.

Despite this progress, many reported markers remain cultivar-specific, some male-associated sequences also occur on autosomes or in both sexes, and epigenetic variation can further complicate interpretation [13]. Thus, although molecular markers are valuable tools for early sex diagnosis and breeding applications, universally reliable markers for predicting sex-expression instability and hermaphroditism remain lacking.

To address this limitation, QTL mapping provides a useful approach for understanding the genetic basis of sex-expression plasticity in *C. sativa*. Faux et al. (2016) identified five distinct QTL positions associated with sex expression in each of three populations, two dioecious and one monoecious [12]. Importantly, a single genomic region marked by 2_299 influenced multiple traits at once, including sex differentiation and the proportion of opposite-sex flowers in both female and male plants, indicating that sex-expression instability is controlled by overlapping quantitative loci rather than a single genetic switch (Table 2). In the monoecious population, QTLs were found not only for the overall proportion of intermediate-sex nodes, but also for how rapidly and at which point along the stem the sex ratio changed, further supporting a multigenic and continuous mode of regulation [34].

Genome-wide association studies (GWAS) have further expanded this understanding at a broader genomic scale, revealing that sex expression in *C. sativa* is a complex trait governed by the interaction of multiple genes rather than a single master locus. Petit et al. (2020) performed a GWAS on 123 hemp accessions across three European environments using approximately 600,000 single nucleotide polymorphism (SNP) markers, identifying two distinct QTLs (QTLSex_det1 and QTLSex_det2) for sex determination (Table 2) [13]. The significant SNP markers defining these QTLs were localized to clusters across three and two genomic scaffolds, respectively. Within the QTLSex_det1 region, the authors identified compelling candidate genes responsible for phytohormone balance, such as a DELLA-family gibberellic acid-insensitive gene (*gai*) and auxin response factors (*arf2* and *arf5*) [13]. This polygenic architecture suggests that sexual plasticity is driven by extensive genetic crosstalk.

In summary, the marker systems and QTLs described above represent valuable tools for cultivar development, early sex identification, and the prioritization of candidate loci underlying sexual plasticity in *C. sativa* [12,13,85,86]. However, their predictive utility for hermaphroditism remains limited because most currently available assays distinguish genetic sex rather than phenotypic instability [85], several published markers exhibit diagnostic or reproducibility limitations [86], and sex-expression instability is additionally influenced by genetic background, environmental stress, phytohormonal regulation, and nutritional conditions [12,13].

## 6. Conclusions

Hermaphroditism in *C. sativa* reflects the highly plastic nature of sex expression in this species and remains a major biological and commercial challenge. From an evolutionary perspective, hermaphroditic or mixed-sex expression may also function as a form of reproductive assurance under pollen limitation or ecological stress, consistent with broader angiosperm evidence that aridity and resource limitation can influence sexual-system variation, reproductive allocation, and mating dynamics [16]. Current evidence indicates that sex phenotype is shaped not only by chromosomal background, but also by hormonal regulation, treatment regime, developmental stage, and environmental conditions. Ethylene- and gibberellin-related pathways are central to this process, with silver-based ethylene inhibitors, particularly STS, providing the most effective means of induced masculinization. At the same time, unintended hermaphroditic expression poses a serious risk in cannabinoid-focused production because even limited pollen release can reduce floral quality, cannabinoid yield, and crop uniformity. Although visual inspection remains the primary management tool, emerging spectroscopic and molecular approaches offer promising support for earlier and more reliable detection.

Although hermaphroditism is commercially undesirable in cannabinoid-focused sinsemilla production, it should not be regarded solely as a pathological or defective trait. Under natural or pollen-limited conditions, the development of functional staminate structures on otherwise pistillate plants may provide reproductive assurance by enabling self-fertilization in the absence of outcross pollen. Consequently, the same sexual plasticity that may enhance reproductive success under environmental constraints becomes economically detrimental in controlled production systems, where unintended pollen release can induce seed formation and reduce floral quality.

Further research is needed to resolve the genetic and physiological basis of sex instability and to develop cultivars and production strategies that minimize hermaphroditic risk while supporting stable, high-value cannabis production.

## Figures and Tables

**Table 2:** Quantitative trait loci (QTLs) relevant to sexual plasticity in *Cannabis sativa* L.

Method	Population	QTL ^(1)^ Adjacent Marker IDs or Scaffold	Description	Reference
Linkage mapping	Dioecious (Group 1)	2_299, 5_323	Sex differentiation	[12]
		2_299, 6_337	Female flower ratio of male plant	
		2_299, 6_243, 6_253	Male flower ratio of female plant	
	Dioecious (Group 2)	2_299, 1_220, 1_318	Sex differentiation	
		1_62, 1_72	Female flower ratio of male plant	
	Monoecious	1_106	Monoecy degree	
		4_121, 6_215	Flower ratio by plant height	
		4_241, 1_149	Node of maximum variation in sex expression	
Genome-wide association study	Dioecious and Monoecious	Scaffold 21,799, Scaffold 32,801, Scaffold 46,170	Contains genes balancing auxin and gibberellic acid	[13]
		Scaffold 9529, Scaffold 11,637	Contains genes related to flowering time and sex determination	

Markers, and locations follow the original labels from the source text. ^(1)^ QTL: quantitative trait locus.

## Data Availability

No new data were created or analyzed in this study.

## Abbreviations

The following abbreviations are used in this manuscript:

1-MCP	1-Methylcyclopropene
ABA	Abscisic Acid
ACS	1-Aminocyclopropane-1-Carboxylate Synthase
AFLP	Amplified Fragment Length Polymorphism
AVG	Aminoethoxyvinylglycine
bp	Base Pair
CBD	Cannabidiol
CBDA	Cannabidiolic Acid
CSP-1	*Cannabis sativa* L. Subtelomeric Repeat Probe
FISH	Fluorescent In Situ Hybridization
GA	Gibberellic Acid
GA_3_	Gibberellic Acid A3
GA_4+7_	Gibberellic Acids A4 and A7
GA_7_	Gibberellic Acid A7
GA_9_	Gibberellic Acid A9
GWAS	Genome-Wide Association Study
IAA	Indole-3-Acetic Acid
MADC	Male-Associated DNA from Cannabis
Mb	Megabase pair
QTL	Quantitative Trait Locus
RAPD	Random Amplified Polymorphic DNA
SDR	Sex-Determining Region
SNP	Single-Nucleotide Polymorphism
STS	Silver Thiosulfate
SuF	Suppressor of Female Development
THC	Tetrahydrocannabinol
THCA	Tetrahydrocannabinolic Acid
Δ^9^-THC	Delta−^9^-Tetrahydrocannabinol
